# Genetics of recurrent pregnancy loss among Iranian population

**DOI:** 10.1002/mgg3.891

**Published:** 2019-07-30

**Authors:** Meysam Moghbeli

**Affiliations:** ^1^ Medical Genetics Research Center Mashhad University of Medical Sciences Mashhad Iran

**Keywords:** abortion, genetic, Iran, marker, miscarriage, recurrent pregnancy loss

## Abstract

**Background:**

Recurrent pregnancy loss (RPL) is one of the most common reproductive disorders which is defined as the occurrence of recurrent miscarriage before 24 weeks of gestation and is observed among 1%–5% of women.

**Methods:**

Various factors are associated with RPL such as immunological disorders, maternal age, obesity, alcohol, chromosomal abnormality, endocrine disorders, and uterine abnormalities. About half of the RPL cases are related with chromosomal abnormalities. Therefore, RPL genetic tests are mainly limited to karyotyping. However, there is a significant proportion of RPL cases without any chromosomal abnormalities that can be related to the single‐gene aberrations. Therefore, it is required to prepare a diagnostic panel of genetic markers besides karyotyping.

**Results:**

In the present review, we have summarized all the significant reported genes until now which are associated with RPL among Iranian women. We categorized all the reported genes based on their cellular and molecular functions in order to determine the molecular bases of RPL in this population.

**Conclusion:**

This review paves the way of introducing a population‐based diagnostic panel of genetic markers for the first time among Iranian RPL cases. Moreover, this review clarifies the genetic and molecular bases of RPL in this population.

## 1 INTRODUCTION

**Figure 1:**
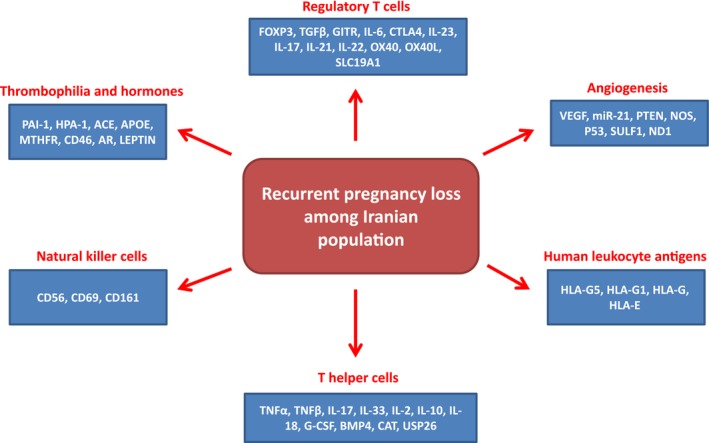
Cellular and molecular processes which are involved in recurrent pregnancy loss among Iranian population

Pregnancy loss is one of the most common disorders during pregnancy which is observed among 12%–15% of women (Zinaman, Clegg, Brown, O'Connor, & Selevan, 1996). The early pregnancy losses also involve about 17%–22% of pregnancies (Ellish et al., 1996). Pregnancies are mainly lost before implantation and next menses which are not clinically diagnosed (Jauniaux & Burton, 2005). About 25% of couples experience at least one sporadic early pregnancy loss (Casikar, Reid, Rippey, & Condous, 2012; Jurkovic, Overton, & Bender‐Atik, 2013). The rate of pregnancy loss decreases to 2.8% after 10–13 weeks of implantation (Pandya, Snijders, Psara, Hilbert, & Nicolaides, 1996). Miscarriage before 20 weeks of gestation is called recurrent pregnancy loss which is observed among 1%–3% of females (Redecha, van Rooijen, Torry, & Girardi, 2009; Yang et al., 2012). Early pregnancy loss is a failed pregnancy prior to 10 weeks of gestation and includes peri‐implantation loss, ectopic pregnancy, pre‐embryonic loss, and embryonic loss (Jauniaux & Burton, 2005; Jurkovic et al., 2013). Chromosomal abnormalities are common problems among the cases with miscarriage in the first trimester (De Braekeleer & Dao, 1990; Goddijn & Leschot, 2000). Aneuploidy and polyploidy involve 86%–91% of chromosomal disorders. Chromosomal deletions, translocations, inversions, and duplications are also important structural changes related to the pregnancy loss (De Braekeleer & Dao, 1990). Chromosomal mosaicism is also associated with 8% early miscarriages (Goddijn & Leschot, 2000; van den Berg, van Maarle, van Wely, & Goddijn, 2012). Chromosomal abnormalities can be related to various molecular reasons such as meiotic homologous recombinations (Sazegari et al., 2014). Moreover, beside the chromosomal and genetic disorders, miscarriages can be caused by several other reasons such as inherited uterine abnormalities, thrombophilia, natural killer (NK) cell dysfunction, abnormal *HLA‐G* expression, diabetes, thyroid disorder, alcohol, smoking, maternal age, and socioeconomic conditions (Larsen, Christiansen, Kolte, & Macklon, 2013; Zlopasa, Skrablin, Kalafatic, Banovic, & Lesin, 2007). A positive history of miscarriage is also associated with pregnancy loss incidence in which the ratio is increased by 2%–3% after first miscarriage (Stirrat, 1990). However, there is still a significant ratio of pregnancy losses without a clear reason. Since there is not a clear panel of genetic markers for the miscarriage screening among Iranian women, we summarized all the significant reported genes until now (Table 1) in the present review. For the first time we categorized all reported genes based on their functions to clarify the molecular overview of this complication among Iranians (Figure 1).

**Table 1:** All the genetic factors associated with recurrent pregnancy loss among Iranian patients

Study (et al)	Year	Gene	Population	Results
Sazegari	2014	SYCP3	100 cases	Polymorphism was correlated with RPL risk
			100 controls	
Naderi‐Mahabadi	2015	FOXP3	195 cases	Polymorphism was correlated with RPL risk
			101 controls	
Nasiri	2016	IL−6 and CTLA−4	120 cases	Polymorphism was correlated with RPL risk
			120 controls	
Rasti	2016	IL−6	121 cases	Polymorphism was correlated with RPL risk
			121 controls	
Tavakoli	2011	IL−6	8 cases	IL−6 underexpression following vitamin D treatment
			8 controls	
Rasti	2016	CTLA−4	120 cases	Polymorphism was correlated with RPL risk
			120 controls	
Saifi	2016	CTLA−4, GITR, IL−10	20 cases	CTLA−4 and GITR underexpression
			20 controls	IL−10 overexpression
Saifi	2014	IL−6, IL−23, IL−17, FOXP3, TGF‐β	20 cases	IL−6, IL−23, and IL−17 overexpression
			20 controls	FOXP3 and TGF‐β underexpression
Roomandeh	2018	IL−17, IL−21, IL−22, TGF‐β	46 cases	Higher serum levels of IL−17, IL−21, and IL−22 Lower serum levels of TGF‐β
			28 controls	
Rahmani	2019	OX40	40 cases 40 controls	Overexpression
Mohtaram	2016	SLC19A1	147 cases 150 controls	Polymorphism was correlated with RPL risk
Rezaei	2002	TNF‐α, TNF‐β, and IL−2	92 cases 40 controls	Higher serum levels
Aboutorabi	2018	TNF‐α	65 cases 65 controls	Polymorphism was correlated with RPL risk
Bahadori	2014	IL−10	85 cases 104 controls	Polymorphism was correlated with RPL risk
Kamali‐Sarvestani	2005	IL−10	139 cases 143 controls	Polymorphism was correlated with RPL risk
Soheilyfar	2018	IL−18, IL−33	300 cases 300 controls	Polymorphism was correlated with RPL risk
Najafi	2014	IL−17	85 cases 85 controls	Polymorphism was correlated with RPL risk
Nasiri	2018	G‐CSF	122 cases 140 controls	Polymorphism was correlated with RPL risk
Mazdapour	2019	BMP4	70 cases 100 controls	Polymorphism was correlated with RPL risk
Sabet	2014	CAT	105 cases 90 controls	Polymorphism was correlated with RPL risk
Asadpor	2013	USP26	72 cases	Mutation
Amirchaghmaghi	2015	VEGF	10 cases 6 controls	Higher serum levels
Hashemi	2018	VEGF	50 cases 50 controls	Polymorphism was correlated with RPL risk
Karami	2018	miR−21, PTEN	25 cases 25 controls	miR−21 underexpression PTEN overexpression
Azani	2017	eNOS	130 cases 110 controls	Polymorphism was correlated with RPL risk
Firouzabadi	2009	P53	167 cases 32 controls	Polymorphism was correlated with RPL risk
Zahraei	2014	SULF1	100 cases 100 controls	Polymorphism was correlated with RPL risk
Colagar	2013	ND1	33 cases 100 controls	Polymorphism was correlated with RPL risk
Aarabi	2011	PAI−1	63 cases 114 controls	Polymorphism was correlated with RPL risk
Khosravi	2014	PAI−1	595 cases 100 controls	Polymorphism was correlated with RPL risk
Shakarami	2015	PAI−1	100 cases 100 controls	Polymorphism was correlated with RPL risk
Jeddi‐Tehrani	2011	PAI−1	100 cases 100 controls	Polymorphism was correlated with RPL risk
Karami	2018	HPA−1	110 cases 110 controls	Polymorphism was correlated with RPL risk
Fazelnia	2016	ACE	100 cases 100 controls	Polymorphism was correlated with RPL risk
Asgari	2013	APOE	81 cases 81 controls	Polymorphism was correlated with RPL risk
Poursadegh Zonouzi	2014	APOE	100 cases 100 controls	Polymorphism was correlated with RPL risk
Jeddi‐Tehrani	2011	MTHFR	100 cases 100 controls	Polymorphism was correlated with RPL risk
Farahmand	2016	MTHFR	330 cases 350 controls	Polymorphism was correlated with RPL risk
Abdi‐Shayan	2016	CD46	141 cases 153 controls	Polymorphism was correlated with RPL risk
Hashemi	2017	HLA‐G	93 cases 93 controls	Polymorphism was correlated with RPL risk
Arjmand	2016	HLA‐G	200 cases 200 controls	Polymorphism was correlated with RPL risk
Arjmand	2016	HLA‐G	117 cases 117 controls	Polymorphism was correlated with RPL risk
Shobeiri	2015	HLA‐G1	30 cases 30 controls	Underexpression
Fotoohi	2016	HLA‐E	200 cases	Polymorphism was correlated with RPL risk
Ghafourian	2014	CD69 and CD161	43 cases 43 controls	CD69 and CD161 overexpressions
Jahaninejad	2013	AR	85 cases 85 controls	Polymorphism was correlated with RPL risk
Saeed	2010	Leptin	81 cases	Higher levels of serum leptin

## 2 REGULATORY T CELLS (TREGS)

Normally the immune system should be able to detect self from nonself antigens. Tregs are a subpopulation of immune cells which prevent the self‐reactivity of immune system. Tregs have an important role in tolerating the fetal–maternal interface during pregnancy (Zenclussen et al., 2006). A decreased number of CD4+ CD25+ Tregs has been observed in recurrent spontaneous abortion (RSA) cases (Yang et al., 2008) which can be associated with *FOXP3* (OMIM: 300292) downregulation as an essential regulator of CD4+ CD25+ Tregs development and function through *NFAT* (Mei, Tan, Chen, Chen, & Zhang, 2010; Wu et al., 2006). It has been shown that there were high frequencies of −924A/G and −20G/A SNPs in *FOXP3* among a subpopulation of Iranian RSA subjects. The 924A/G is located in *GATA‐3* binding site and the A allele is much required for the promoter binding. Therefore, G allele and G/G can be associated with abortion through suppression of Th2‐immune response (Naderi‐Mahabadi et al., 2015). Treg cells prepare a tolerance in endometria against the fetus for an efficient implantation using *CTLA‐4* (OMIM: 123890) as a negative regulator of T cells (Read et al., 2006; Zenclussen, 2006). *CTLA‐4* suppresses IL‐6 through upregulation of *TGF‐ β* which results in the differentiation of naïve CD4+T cells to Tregs (Perrier d'Hauterive et al., 2004). *IL‐6* (OMIM: 147620) cytokine also regulates the balance between T helper (Th)‐17 and Treg cells through induction and inhibition of Th‐17 and Treg differentiations, respectively (Bettelli et al., 2006; Gardner, Jeffery, & Sansom, 2014). It has been reported that there was a protective role of G allele in *CTLA‐4*+49A/G polymorphism against RPL among a group of Iranian cases. Moreover, there was a significant correlation between *IL‐6* 634C/G polymorphism and RPL in which the G allele was associated with >5 times increase in RPL risk. Therefore, they concluded that the 634C/G variant of *IL‐6* and the +49A/G SNP of *CTLA‐4* can be introduced as RPL risk factors among Iranian women (Nasiri & Rasti, 2016; Rasti, Nasiri, & Kohan, 2016). Another group also reported that the +49 G allele decreased RSA risk among Iranian cases (Rasti & Nasiri, 2016). Treg cells are characterized by several markers such as *GITR* and *CTLA‐4* (Corthay, 2009). *IL‐10* (OMIM: 124092) is also a Treg cell‐related cytokine that regulates the IFN‐g and TNF cytokines (Kwak‐Kim et al., 2003). It has been reported that there were significantly lower levels of *CTLA‐4* and *GITR* expressions among a sample of Iranian RSA cases compared with controls. Moreover, they observed increased *IL‐10* expression in RSA compared with control subjects (Saifi et al., 2016). *IL‐6* and *TGF‐β* (OMIM: 190180) are critical factors during the differentiation of CD4+T cells into Th17 and Treg cells in which the presence of *IL‐6* and *TGF‐β *induces Th17 differentiation, whereas the presence of *TGF‐β* results in Treg cells differentiation. Higher and lower expressions of *IL‐6* and *TGF‐β* respectively have been observed among cases with pregnancy loss (Arruvito, Billordo, Capucchio, Prada, & Fainboim, 2009; Arruvito, Sotelo, Billordo, & Fainboim, 2010; Schumacher et al., 2009). It has been shown that there were significant higher expressions of *IL‐6*, *IL‐23*, and *IL‐17* among a subpopulation of Iranian cases with RPL in comparison with normal nonpregnant subjects. Moreover, the RPL cases had lower levels of *TGF‐β *and *FOXP3* expressions compared with normal nonpregnant subjects (Saifi et al., 2014). *IL‐17* (OMIM: 603149) is secreted by Th17 cells which are related to the Treg cells and regulate the immunological rejection of foreign tissues (McGeachy & Cua, 2008). Therefore, Th17/Treg imbalance can result in pregnancy loss (Sereshki et al., 2014). It has been reported that there were significant higher serum levels of Th17‐related cytokines such as *IL‐17*, *IL‐21*, and *IL‐22* among a subpopulation of Iranian URSA cases compared with normal nonpregnant subjects. In contrast, they reported significant lower levels of Treg‐associated cytokine (*TGF‐β*) in URSA compared with controls (Roomandeh et al., 2018). The costimulatory and coinhibitory signals also regulate cell‐mediated immunity (Liu, Almo, & Zang, 2016). OX40 is a cell surface costimulatory factor expressed by activated CD4 and CD8 T cells which regulate NF‐κB, PI3K/Akt, and calcium/NFAT signaling pathways (So, Song, Sugie, Altman, & Croft, 2006). Moreover, *OX40* downregulates *FOXP3* as the master regulator of Treg cells (Hori, Nomura, & Sakaguchi, 2003). It has been reported that there were higher levels of *OX40* and *OX40L* expressions among a group of Iranian RSA cases in comparison with the healthy cases. They introduced the elevated serum *OX40L* levels as a risk factor of RSA (Rahmani, Hadinedoushan, & Ghasemi, 2019). Folate is involved in different biological processes such as Treg cell maintenance and fetal development (Kim et al., 2013; Kunisawa, Hashimoto, Ishikawa, & Kiyono, 2012). Solute Carrier Family 19 (SLC19A1; OMIM: 600424) is a membranous transporter of 5‐methyltetrahydrofolate. It has been observed that there was a significant correlation between −43T>C polymorphism of *SLC19A1* and RPL among a subpopulation of Iranian cases. Moreover, they introduced certain *SLC19A1* haplotypes as RPL risk factors (Mohtaram et al., 2016).

## 3 T‐HELPER CYTOKINES

T helper (Th) or CD4+cells are a class of T cells mainly involved in adaptive responses. Th cells differentiate into the Th1 and Th2 subtypes which are associated with cell‐mediated and humoral immune responses, respectively. Although, successful pregnancy is associated with Th2‐related cytokines, there is reduced Th1 cytokine production during pregnancy (Clifford, Rai, Watson, & Regan, 1994). Th1 cell responses are determined by the presence of *TNF*, *IFN‐γ*, *IL‐2*, and *IL‐12* cytokines, whereas the Th2 response is defined by *IL‐5*, *IL‐6*, *IL‐4*, and *IL‐10* (Raghupathy et al., 2000). It has been observed that there was a correlation between Th1 cytokines and miscarriages among a sample of Iranian RSA cases in which the RSA cases had significant higher serum *TNF‐α*, *TNF‐β*, and *IL‐2* levels compared with control cases (Rezaei & Dabbagh, 2002). Another group has reported that there were also significant correlations between −863C/A and −238G/A variants of *TNF‐α* (OMIM: 191160) and RPL among a sample of Iranian cases. The −308G allele was also a protective factor against spontaneous abortion (Aboutorabi et al., 2018). A balanced cytokine production between Th1 and Th2 cells is required for a successful pregnancy (Chaouat et al., 2002). Although the increased levels of pro‐inflammatory cytokines are correlated with pregnancy termination, *IL‐10* as an anti‐inflammatory cytokine inhibits Th1‐mediated cellular reactions which are important for the preservation of pregnancy (Choi & Kwak‐Kim, 2008; Raghupathy et al., 1999). *IL‐10* is an inhibitor of Th1‐mediated cellular responses through suppression of *IFN‐ γ* and *TNF* cytokines. It has been reported that the RM cases had significantly higher frequency of the *IL‐10* –592 A/C genotype in comparison with controls. Moreover, there was also a correlation between *IL‐10* –819 C/T polymorphism and RM among a sample of Iranian subjects (Bahadori et al., 2014). It has been observed that there was a significant higher frequency of the *IL‐10* –592 CC genotype in RPL in comparison with the healthy cases among a subpopulation of Iranian subjects. They showed that the 592 CC genotype carriers secrete lower levels of *IL‐10* (Kamali‐Sarvestani, Zolghadri, Gharesi‐Fard, & Sarvari, 2005). *IL‐18* (OMIM: 600953) is produced by a wide range of immune and nonimmune cells which are involved in regulation of Th1 and Th2 differentiations (Blom & Poulsen, 2012). *IL‐33* (OMIM: 608678) is also produced by endothelial cells and is associated with Th2 activation (Balato et al., 2014; Lefrancais et al., 2014). It has been reported that there was a significant correlation between *IL‐18* (rs1946518) polymorphism and RPL in which the CC genotype can be a RPL risk factor among a subpopulation of Iranian cases. Moreover, they also observed the GA genotype of *IL‐33* (rs1929992) polymorphism as a RPL risk factor among Iranian subjects (Soheilyfar et al., 2018). *IL‐17* (OMIM: 603149) is produced by Th‐17 cells following *IL‐23* induction. It has been observed that there was a significant different frequency of *IL‐17F* (rs763780) gene polymorphism between a group of Iranian RPL and control subjects which showed that this polymorphism can be correlated with a high RPL risk in this population (Najafi, Hadinedoushan, Eslami, & Aflatoonian, 2014). Granulocyte colony stimulating factor (*G‐CSF*; OMIM: 138970) is a glycoprotein observed in endothelial cells and macrophages and is associated with upregulation of *IL‐4* and *IL‐10* anti‐inflammatory cytokines. Moreover, it is involved in shifting the Th1/2 balance toward the Th2 responses (Boneberg & Hartung, 2002; Mannon et al., 2009). It has been reported that there were significant different frequencies of CT and T allele (TT+CT) genotypes of the rs1042658 between a sample of Iranian RPL cases and controls that introduced this polymorphism as a probable RPL risk factor among Iranians (Nasiri & Jahangirizadeh, 2018). *BMP4* (OMIM: 112262) as a ligand of the *TGFβ* family activates the *SMAD* transcription factors and can be associated with regulation of early ovarian follicle development (Nilsson & Skinner, 2003). Moreover, the canonical BMP signaling is involved in the activation of CD4 T cells (Martinez et al., 2015). It has been shown that there was a higher frequency of A allele of *BMP4* (rs121912765) polymorphism among a sample of Iranian RSA patients in comparison with controls who showed this polymorphism as a RSA risk factor in Iran (Mazdapour, Dehghani Ashkezari, & Seifati, 2019). Oxidant and antioxidant balance has a critical role in the preservation of normal physiological conditions during a successful pregnancy. Reactive oxygen species (ROS)‐related damages result due to pregnancy complications because of the lack of antioxidants (Wang, Walsh, Guo, & Zhang, 1991). ROS are involved in T‐cell regulation in which the high ROS levels prolong Th‐2‐mediated immune responses, whereas reduced levels induce the Th‐1 and Th‐17 differentiation (Kaminski et al., 2010; Yarosz & Chang, 2018). The catalase (*CAT*; OMIM: 115500) is a pivotal antioxidant enzyme that functions as a protector against the ROS‐related cell damage through conversion of hydrogen peroxide to oxygen and water (Rohrdanz & Kahl, 1998). It has been observed that there was a significant correlation between *CAT* 262C/C genotype and increased spontaneous abortion susceptibility among a subpopulation of Iranian cases (Sabet, Salehi, Khodayari, Zarafshan, & Zahiri, 2014). Ubiquitin‐specific protease 26 (*USP26*; OMIM: 300309) is one of the members of deubiquitinating enzymes (DUB) involved in the regulation of cell growth, differentiation, and tumorigenesis (Amerik & Hochstrasser, 2004; Glickman & Ciechanover, 2002). *USP26* regulates the *TGF‐β* signaling through stabilization of *SMAD7* (Kit Leng Lui et al.., 2017). *TGF‐β* also has pleiotropic effects on adaptive immunity and regulation of CD4+T‐cell responses (Travis & Sheppard, 2014). It has been shown that the *USP26* gene mutations can be associated with infertility and RPL among a subpopulation of Iranian males and females, respectively (Asadpor et al., 2013).

## 4 ANGIOGENESIS

Angiogenesis is a critical physiological process during a successful pregnancy. *VEGF* (OMIM: 192240) is an angiogenic cytokine which increases the vascular permeability and is involved in the regulation of proliferation and differentiation of endothelial cells (Dvorak, Brown, Detmar, & Dvorak, 1995; Ferrara, Houck, Jakeman, & Leung, 1992). Various factors upregulate the *VEGF* expression such as hypoxia, *EGF*, *TGF‐β*, and *IL‐1β* (Ferrara & Davis‐Smyth, 1997). It has been reported that there was significant high levels of serum *VEGF* among a subpopulation of Iranian URSA cases (Amirchaghmaghi et al., 2015). Moreover, another group has reported that the 18‐bp ins/del polymorphism in *VEGF* significantly increased the risk of RSA in a sample of southeast Iranian cases (Hashemi et al., 2018). Micro‐RNAs are a class of noncoding RNAs involved in posttranscriptional regulation through mRNA degradation or block of translation (Bartel, 2009). They have key roles in various reproductive system disorders such as preeclampsia and RM (McCallie, Schoolcraft, & Katz‐Jaffe, 2010). Aberrant angiogenesis is one of the mechanisms correlated with pregnancy loss (Papazoglou et al., 2005). *MiR‐21* (OMIM: 611020) targets PTEN during the regulation of angiogenesis. Moreover, *miR‐21* overexpression activates the ERK and AKT signaling pathways which results in *VEGF* upregulation and increased angiogenesis (Liu et al., 2011). It has been observed that there were *miR‐21* under and PTEN overexpressions among a subpopulation of Iranian RM cases (Karami, Mirabutalebi, et al., 2018). Nitric oxide (NO) is involved in the regulation of many aspects of pregnancy such as fetomaternal angiogenesis and blood circulation which are required for a successful pregnancy (Sladek, Magness, & Conrad, 1997; Suryanarayana et al., 2006). Therefore, reduced NO production can result in aberrant placental perfusion and pregnancy loss (Su, Lin, & Chen, 2011). Nitric oxide synthases (NOSs) are responsible for the generation of soluble NO from l‐arginine (Shin et al., 2010). It has been observed that there were significant higher frequencies of *eNOS* −786 T>C variants and eNOS −786C alleles among a subpopulation of Iranian RPL cases compared with healthy subjects in which the *eNOS* −786C allele increased the risk of early pregnancy loss (Azani et al., 2017). Since the normal pregnancy requires sufficient fetal placental circulation, reduced vascular development can be correlated with early pregnancy loss (Reynolds & Redmer, 2001). Normally there is a low rate of apoptosis during the first trimester in placenta, whereas it has a raising ratio as gestation progresses (Smith, Baker, & Symonds, 1997). The P53 (OMIM: 191170) is a multifunctional transcription factor regulating cell apoptosis and angiogenesis (Ravi et al., 2000; Yuan et al., 2002). It has been shown that there was a significant correlation between RPL and P53 codon 72 gene polymorphism in which the Pro/Pro cases had higher risk of RPL compared with the Arg/Arg genotype cases among a sample of Iranian subjects (Firouzabadi, Ghasemi, Rozbahani, & Tabibnejad, 2009). Arylendosulfatase (*SULF*; OMIM: 610012) is a heparin sulfatase that releases 6‐O‐sulfate groups from heparin sulfates which change the growth factor binding sites in proteoglycans (Ai et al., 2006). Therefore, SULFs can be associated with angiogenesis and embryogenesis (Dhoot et al., 2001). A group has reported a correlation between *SULF1* (rs6990375G>A) polymorphism and increased risk of recurrent miscarriage among a subpopulation of Iranian patients in which there were higher frequencies of GG and AA homozygous genotypes among patients. Moreover, higher frequency of AG genotype among healthy cases showed a correlation between this genotype and higher chance of successful pregnancy (Zahraei et al., 2014). Mitochondria are the cellular bioenergetic centers that have fundamental role during cell proliferation and development through oxidative phosphorylation and ATP production (Dumollard, Duchen, & Carroll, 2007). This organelle as a cellular oxygen sensor regulates angiogenesis through epithelial proliferation and migration. The NADH dehydrogenase I (*ND1*; OMIM: 516000) is one of the components of NADH dehydrogenase complex, which is the largest complex in the electron transport chain. The T4216C variation of *ND1* has been observed in 30% of a subpopulation of Iranian RPL cases which can be introduced as a polymorphism with secondary effects on RPL (Colagar et al., 2013).

## 5 THROMBOPHILIA

Stable pregnancy requires a balance between maternal coagulation and fibrinolysis which stabilizes the placental basal plate (Buchholz & Thaler, 2003). Thrombophilia is a hypercoagulable state associated with several complications such as thrombotic pregnancy, preeclampsia, and abortion (Kempf Haber & Klimek, 2005). The fibrinolytic system is one of the endogenous defense mechanisms against intravascular thrombosis (Collen & Lijnen, 1986). Fibrinolytic activity can be associated with plasminogen activator inhibitor (PAI) (Lane & Grant, 2000). *PAI‐1* as a tissue plasminogen (t‐PA) inhibitor that has an important role in thrombotic disorders and increased *PAI‐1* concentration can be associated with placental damage through aberration in coagulation and fibrinolysis (Coulam, Wallis, Weinstein, DasGupta, & Jeyendran, 2008). It has been shown that there was a significant higher frequency of *PAI‐1* (4G/4G) polymorphism among Iranian RSA cases compared with healthy subjects (Aarabi et al., 2011; Jeddi‐Tehrani et al., 2011; Khosravi et al., 2014; Shakarami, Akbari, & Zare Karizi, 2015). Fibrinogen is one of the key factors in coagulation process which regulates the platelet aggregation endothelial activity (Voetsch & Loscalzo, 2004). The human platelet antigen‐1 (*HPA‐1*) is a fibrinogen receptor that is associated with platelet activation and thrombosis stimulation (Shattil, 1999). It has been shown that there was a correlation between rs5918 T>C polymorphism of *HPA‐1* and RPL risk in a sample of Iranian subjects in which this polymorphism was mainly observed among RPL cases (Karami, Askari, & Modarressi, 2018). Angiotensin converting enzyme (*ACE*; OMIM: 106180) as a key thrombophilic factor converts angiotensin I to angiotensin II and is associated with platelet aggregation and fibrinolysis. It has been reported that there was a correlation between *ACE* I/D polymorphism and RPL among a sample of Iranian population in which the DD genotype was more frequent in RPL compared with control cases. It was concluded that the *ACE* D allele can increase RPL risk and can be considered as a diagnostic factor among Iranian RPL cases (Fazelnia, Farazmandfar, & Hashemi‐Soteh, 2016). Apo E (OMIM: 107741) polymorphism is another thrombophilic factor highly expressed in liver and brain (Wernette‐Hammond et al., 1989) which is correlated with the metabolism of cholesterol and triglyceride through LDL receptors (Mahley, 1988). It is associated with different immunological processes such as T‐cell proliferation and NK cell activation. *APOE* has three allelic variants including E2–4. It has been reported that there was a significant higher frequency of allele E4 among a subpopulation of Iranian RPL patients compared with non‐RPL cases (Asgari, Akbari, Zare, & Babamohammadi, 2013; Poursadegh Zonouzi, Farajzadeh, Bargahi, & Farajzadeh, 2014). Hypercoagulable state increases the thrombophilia which can be maintained by factors involving in coagulation system and folate metabolism (Blanco‐Molina et al., 2007). Irregular folate pathway is associated with hyperhomocysteinemia which causes endothelial damage via elevated oxidative stress (Sen, Mishra, Tyagi, & Tyagi, 2010). Methylenetetrahydrofolate reductase (*MTHFR*; OMIM: 607093) is involved in methionine formation from homocysteine. The Iranian RPL cases had significantly higher frequencies of *MTHFR* 677C ⁄T and 1298A ⁄C polymorphisms compared with the control group (Farahmand et al., 2016; Jeddi‐Tehrani et al., 2011). Aberrant complement system is one of the immunologic factors involved in RSA. *CD46* (OMIM: 120920) is a transmembrane glycoprotein which functions as a complement factor I cofactor to suppress C3 convertase complex and maintains complement activation (Liszewski, Post, & Atkinson, 1991). The *CD46* gene mutations have been reported in various disorders such as preeclampsia and RSA (Lokki, Aalto‐Viljakainen, Meri, Laivuori, & Finnpec, 2015; Risk, Flanagan, & Johnson, 1991). It has been observed that there was a correlation between *CD46* IVS1‐1724 C>G polymorphism and RSA risk in a sample of Iranian cases (Abdi‐Shayan, Monfaredan, Moradi, Rajaii Oskoui, & Kazemi, 2016).

## 6 HUMAN LEUKOCYTE ANTIGENS

Human leukocyte antigens (HLAs) encode the major histocompatibility complex proteins as the regulators of immune system. The HLA system helps immune system to discriminate between self and nonself cells. The HLA expression at feto‐maternal interface can be associated with a successful pregnancy (Ellis, Palmer, & McMichael, 1990; Kovats et al., 1990). *HLA‐G* (OMIM: 142871) is normally expressed in several locations such as fetal trophoblasts, pancreatic islets, and endothelial precursors (Carosella & LeMaoult, 2011). *HLA‐G* protects fetal trophoblast cells toward the maternal uterine NK cells during pregnancy (Abediankenari, Farzad, Rahmani, & Hashemi‐Soteh, 2015). It has been reported that there was a significant correlation between *HLA‐G* 3142G>C and 14‐bp ins/del polymorphisms and RSA susceptibility among a group of Iranian cases (Hashemi et al., 2017). The 14‐bp deletion/insertion polymorphism in *HLA‐G* is correlated with the regulation of *HLA‐G* expression. It has been reported that there was a higher frequency of heterozygote +14‐bp in a group of Iranian cases with recurrent miscarriages compared with fertile controls (Arjmand & Samadi, 2016). Another group reported that there was a significant association between HLA‐G*0105N alleles and lower serum *HLA‐G* levels which increased the risk of RSA among a subpopulation of Iranian cases (Arjmand, Ghasemi, Mirghanizadeh, & Samadi, 2016). The *HLA‐G1* and *HLA‐G5* were decreased among Iranian abortion threatened cases. The abortion threatened cases had significant lower levels of *HLA‐G1* expression compared with control cases. Moreover, there was a direct association between *HLA‐G1* and *HLA‐G5* expression and *IL‐10* levels and a converse association between NK cell numbers and these cytokines which introduced uterine NK, *HLA‐G1*, and *HLAG5* as key factors in fetal maintenance during pregnancy among Iranians (Shobeiri et al., 2015). *HLA‐E* (OMIM:143010) is another member of the HLA proteins involved in feto‐maternal tolerance through interaction with CD94/NK G2A complex which has a critical role in NK cell suppression. It has been observed that there was higher frequency of *HLA‐E 0101* polymorphism among a sample of Iranian RSA cases compared with controls, whereas *HLA‐E 0103* was more frequent among controls. Moreover, the *HLA‐E0101/0103* heterozygous genotype was correlated with fetus maintenance among Iranians (Fotoohi, Ghasemi, Mirghanizadeh, Vakili, & Samadi, 2016).

## 7 NATURAL KILLER (NK) CELLS

Natural killer (NK) cells are cytotoxic lymphocytes associated with maternal immune system suppression. They are the most abundant immune cells in uterine implantation site and act as the first cellular immune defense mechanism. NK cells are classified into CD16+CD56dim and CD16−CD56bright NK cells (Saito, Nakashima, Myojo‐Higuma, & Shiozaki, 2008). Increased peripheral blood NK cells are associated with higher rate of aberrant implantation following in vitro fertilization (IVF) (Thum et al., 2004). *CD56* is a cell adhesion molecule (NCAM1; OMIM: 116930) that regulates the interaction between NK cells and their target cells (Vivier, Tomasello, Baratin, Walzer, & Ugolini, 2008). High NK cell activities cause trophoblast cell damage and abortion (Kwak‐Kim & Gilman‐Sachs, 2008). It has been shown that there was a significant increase in NK cytotoxicity among a subpopulation of Iranian RSA cases compared with controls. Moreover, the RSA cases had significantly higher percentage of CD56dim cells in comparison with control cases (Karami, Boroujerdnia, Nikbakht, & Khodadadi, 2012). RSA and IVF failure can be associated with immunological deficiencies during interactions between maternal immune cells and fetus. This immunological interaction can be related to NK cells and there is a direct correlation between increased NK cells and placental damage (Moffett‐King, 2002). *CD69* (OMIM: 107273) and *CD161* (OMIM: 602890) are cell surface markers involved in cytokine production and cytotoxicity (Marzio, Mauel, & Betz‐Corradin, 1999; Pozo et al., 2006). It has been observed that there were significantly increased CD69 NK cells among a group of Iranian cases with RAS and IVF failure compared with healthy subjects. Moreover, they observed increased *CD161* expression on NK cells in RSA and IVF failure cases compared with normal cases with successful pregnancy. Therefore, *CD69* and *CD161* overexpression on NK cells can be considered as a risk factor of RSA and IVF failure among Iranians (Ghafourian, Karami, Khodadadi, & Nikbakht, 2014).

## 8 HORMONES

Vitamin D is a lipid‐soluble hormone involved in bone and mineral metabolism by binding with nuclear vitamin D receptor (*VDR*; OMIM: 601769) (Jones, Strugnell, & DeLuca, 1998). Expression of *VDR* and vitamin D hydroxylation enzymes in placenta and decidua show the key role of this hormone in the regulation of the reproduction system (Vigano et al., 2006). Vitamin D3 suppresses and induces the *IL‐12* and *IL‐10* productions respectively in dendritic cells which direct the cytokine profile toward humoral immune response. It has been shown that vitamin D3 has a preventive role in preeclampsia (Halhali et al., 2000). Moreover, it is expressed by a variety of endometrial resident cells, such as epithelial cells, macrophages, and dendritic cells (Vienonen et al., 2004). *IL‐6* has a critical role in suppression of regulatory T‐cell development which are involved in normal pregnancy (Saito, Nakashima, Shima, & Ito, 2010). It has been reported that vitamin D3 is probably involved in the regulation of inflammatory responses which can result in abortion. Moreover, there was a significant decrease in *IL‐6* production by whole endometrial and endometrial stromal cells among Iranian URSA cases following vitamin D3 treatment (Tavakoli et al., 2011). Androgens are essential lipophilic hormones for differentiation of endometrial stromal cells into decidual cells which regulate the embryo implantation and placentation (Guay et al., 2004). Androgen receptor (*AR*; OMIM: 313700) is a nuclear receptor highly expressed in the female reproductive system (Apparao, Lovely, Gui, Lininger, & Lessey, 2002). The *AR*‐G1733A polymorphism has been assessed in a sample of Iranian RSA cases and a correlation between A allele and elevated risk of pregnancy loss was observed (Jahaninejad, Ghasemi, Kalantar, Sheikhha, & Pashaiefar, 2013). Leptin (*LEP*; OMIM: 164160) is a hormone mainly produced by adipocytes and is involved in the energy balance and body weight homeostasis (Zhang et al., 1994). Moreover, leptin is secreted by trophoblasts and has a rising trend of serum levels until the second trimester (Schubring et al., 1997). Placenta can be the main source of maternal leptin, since the leptin levels drops following parturition (Malik et al., 2005). Leptin overexpression can be indirectly correlated with RSA through production of Th1‐associated autoantibodies. It has been observed that there was higher levels of serum leptin among a subpopulation of Iranian recurrent abortion cases in comparison with normal subjects (Saeed et al., 2010).

## 9 CONCLUSIONS

Recurrent pregnancy loss is a serious growing problem among the young couples. Despite various clinical and experimental tests, still there is not an accurate and efficient diagnostic method during the early stages of pregnancy in cases without any known cause. Therefore, it is required to determine targeted genomic methods besides karyotyping, clinical, and pathological examination. In the present review, we summarized all the reported single gene abnormalities among Iranian RPL cases to pave the way of introducing a population‐based panel of genetic markers in this population. We categorized all the reported markers to clarify the molecular bases of RPL. We observed that the majority of reported markers belonged to the regulatory T cells which highlights the role of these immune cells in RPL among Iranian women.

## CONFLICT OF INTEREST

None declared.
